# Supplementary material for: Metabolic fluxes for nutritional flexibility of Mycobacterium tuberculosis
Source: Mol Syst Biol. 2021 May 4;17(5):e10280. doi: 10.15252/msb.202110280 (PMC8094261; doi:10.15252/msb.202110280)
Supplement: Supplementary file 1 — Expanded View Figures PDF [file MSB-17-e10280-s003.pdf]

Expanded View Figures

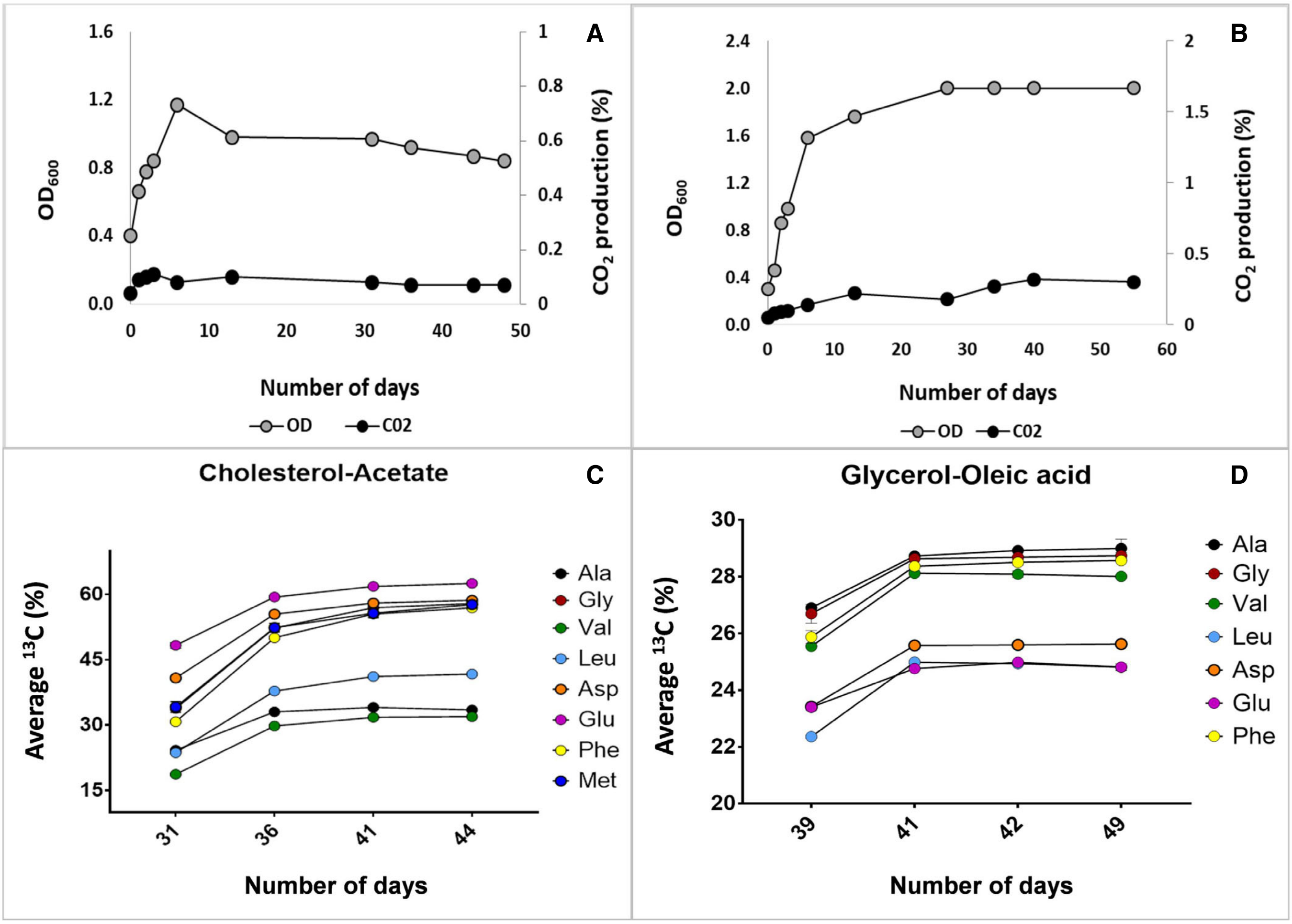

Figure EV1. Metabolic and isotopic steady-state growth of *Mycobacterium tuberculosis*.

A–D Continuous cultures of Mtb were grown in bioreactors with either CHL-ACE or GLY-OLA acid as carbon sources at a dilution rate of 0.01 h^{-1} (t_d = 69 h). Metabolic steady state was achieved after three volume changes as evidenced from by stable cell density (OD_{600}) and CO_2 production rates (A and B). After metabolic steady state was achieved, the feed was replaced with identical media containing ^{13}C-labelled carbon substrates until the culture attained an isotopic steady state as evidenced by steady levels of ^{13}C incorporation into proteinogenic amino acids.

© 2021 The Authors

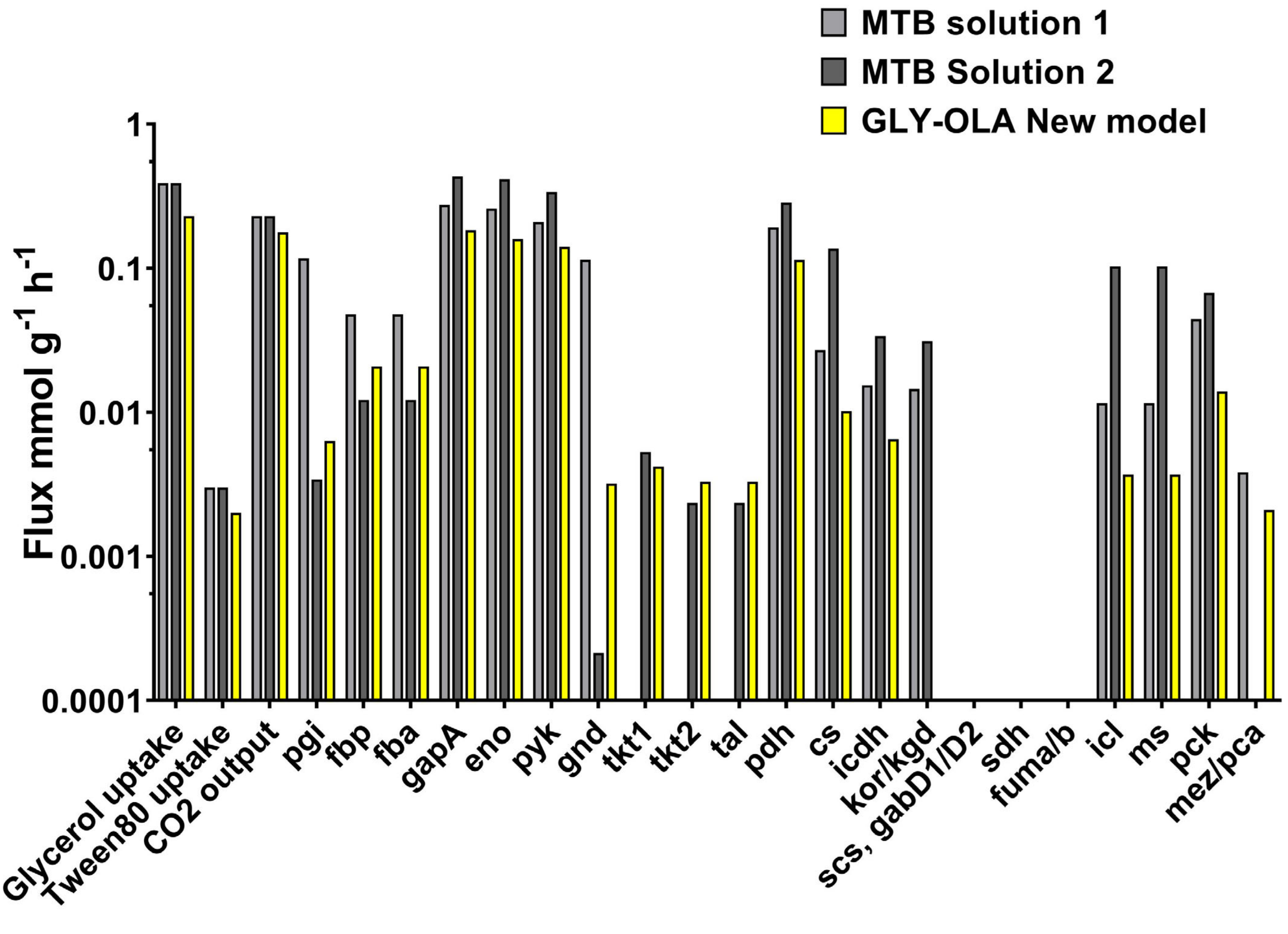

Figure EV2. Absolute flux distributions.

Fluxes measured in this study with the extended metabolic model were compared to the previously measured fluxes in Beste *et al* (2011).

 © 2021 The Authors

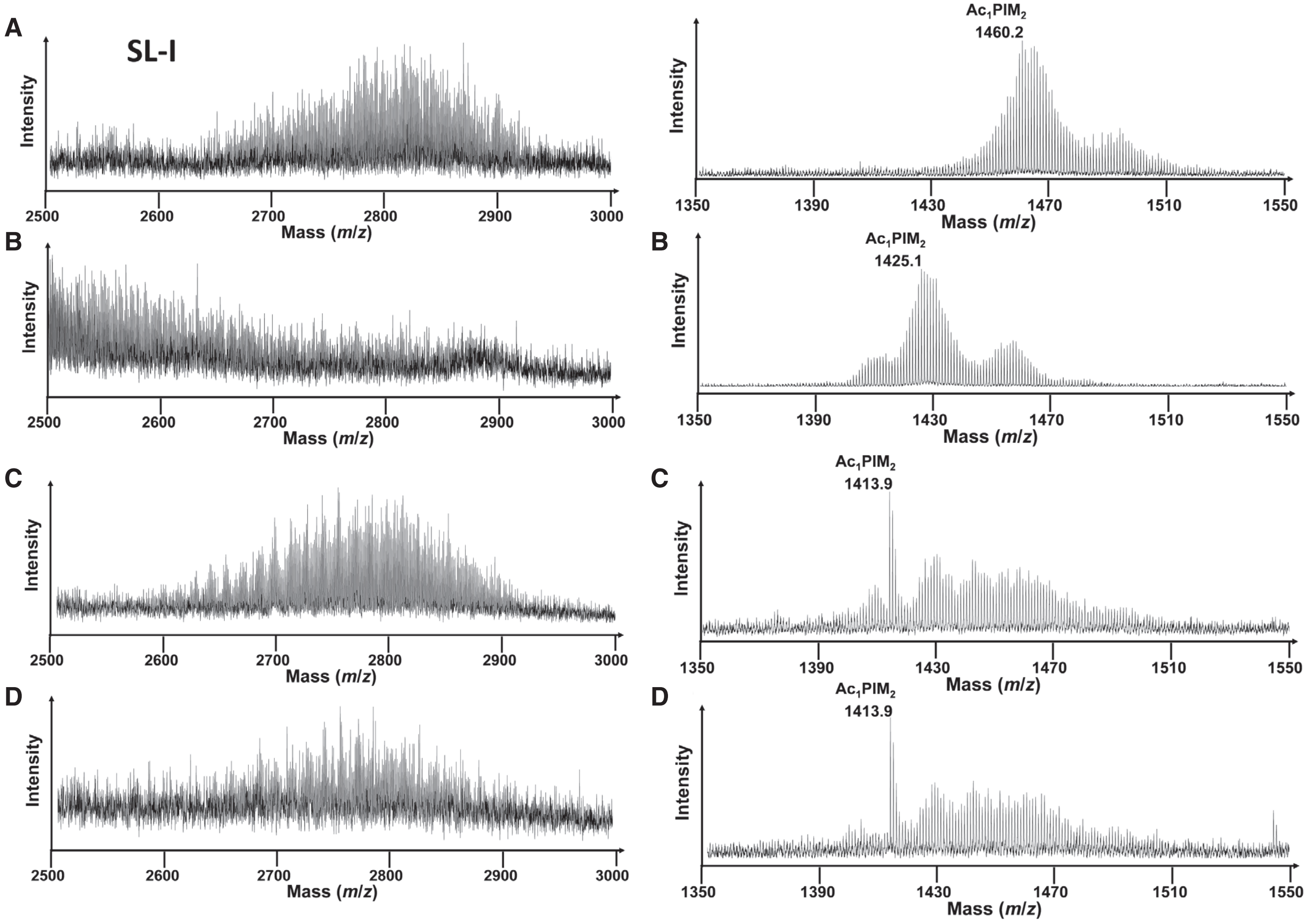

Figure EV3. SL-I and Ac1PIM2 lipid finger printing.

A–D SL-I is observed as a broad set of peaks representing multiple lipoforms corresponding to differing numbers of CH_2 units. Multiple lipoforms of SL-1 (m/z 2500 to m/z 3000) are also indicated SL-I. Mass shifts due to ^{13}C labelling of whole bacteria lipid fingerprint prepared from metabolic and isotopic steady-state chemostat for Mtb grown with [$^{13}C_2$] acetate and unlabelled cholesterol (A), [$^{13}C_3$]-glycerol and unlabelled Tween 80 (B), unlabelled acetate and cholesterol (C) and unlabelled glycerol and Tween 80 (D). Samples taken from the same chemostat at metabolic steady state (A) and (B) and isotopic in stationary state (C) and (D). The resolved mass shifts observed for Ac1PIM2 are shown to indicate significant lipid changes between the four conditions.

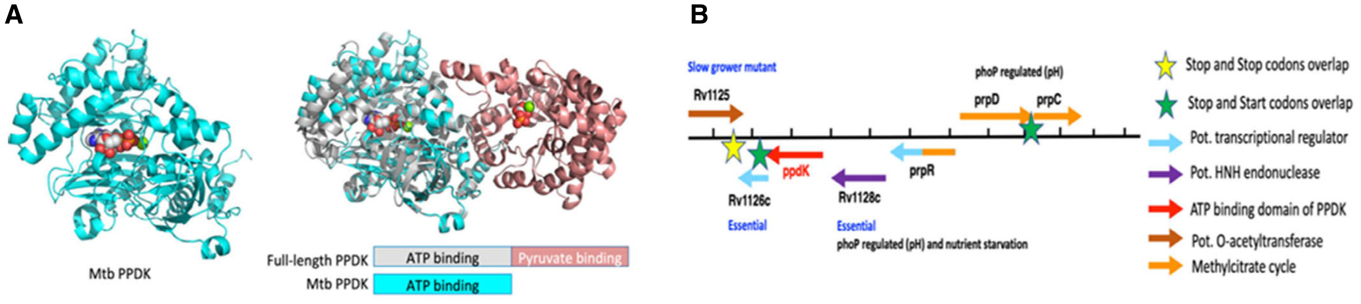

Figure EV4. PPDK structure and genetic organisation in Mtb.

A Left panel: iTasser model of Rv1127c (cyan) with ATP bound. Right panel: Overlay of the full-length PPDK (N-term domain white, C-term salmon with Rv1127c (cyan)) showing that Rv1127c is lacking the PEP/pyruvate-binding domain.

B Mtb genomic organisation of Rv1127c (ppdk, red arrow).

 © 2021 The Authors
